# Applications of Radiolabelled Curcumin and Its Derivatives in Medicinal Chemistry

**DOI:** 10.3390/ijms22147410

**Published:** 2021-07-10

**Authors:** Matteo Mari, Debora Carrozza, Erika Ferrari, Mattia Asti

**Affiliations:** 1 Department of Chemical and Geological Sciences, University of Modena and Reggio Emilia, Via G. Campi 103, 41125 Modena, Italy; matteo.mari96@outlook.it (M.M.); 213134@studenti.unimore.it (D.C.); erika.ferrari@unimore.it (E.F.); 2 Radiopharmaceutical Chemistry Section, Nuclear Medicine Unit, AUSL-IRCCS di Reggio Emilia, Viale Risorgimento 80, 42122 Reggio Emilia, Italy

**Keywords:** curcumin, radionuclides, Alzheimer’s disease, curcuminoids, radioactive labelling

## Abstract

Curcumin is a natural occurring molecule that has aroused much interest among researchers over the years due to its pleiotropic set of biological properties. In the nuclear medicine field, radiolabelled curcumin and curcumin derivatives have been studied as potential radiotracers for the early diagnosis of Alzheimer’s disease and cancer. In the present review, the synthetic pathways, labelling methods and the preclinical investigations involving these radioactive compounds are treated. The studies entailed chemical modifications for enhancing curcumin stability, as well as its functionalisation for the labelling with several radiohalogens or metal radionuclides (fluorine-18, technetium-99m, gallium-68, etc.). Although some drawbacks have yet to be addressed, and none of the radiolabelled curcuminoids have so far achieved clinical application, the studies performed hitherto provide useful insights and lay the foundation for further developments.

## 1. Introduction

Although curcumin has attracted the attention of researchers since its isolation by Lampe in 1918 [1], it was only in the 1990s, after the first reports claiming for its anti-oxidant [2], anti-inflammatory [3], and anticancer [4] effects, that the interest in curcumin medical applications started to grow at a rapid pace. 

Curcumin [(*E,E*)-1,7-bis(4-hydroxy-3-methoxyphenyl)-1,6-heptadiene-3,5-dione] represents the biologically active ingredient of turmeric, a yellow colouring compound found in the rhizomes of *Curcuma longa* L., a plant belonging to the ginger family. As with many naturally occurring molecules, curcumin can be extracted and purified from the dried rhizomes using traditional/green techniques or can be directly synthesised. As pointed out by Zerazion et al. [5], despite expectations, the chemical synthesis of curcumin constitutes the less environmentally impacting procedure to gain highly pure curcumin at a laboratory scale. This outcome may be attributed to the higher reaction yield in comparison to the extraction one. The worldwide known synthesis of curcumin was first proposed by J.J. Pabon in 1964 [6], and it is based on the reaction of protected acetylacetone with vanillin or a derivative thereof. Protection of the β-diketo group is obtained by forming a boron acetylacetonate complex to activate the methyl groups for the aldol condensation. One of the most interesting chemical features of curcumin is the keto-enolic moiety that is probably the driving force for its reactivity/(in)stability, and consequently, ADME (absorption, distribution, metabolism and excretion) properties. Indeed, the β-keto-enol group undergoes a tautomeric equilibrium [7,8] that is strongly solvent- and pH-dependent. The keto-enol form is stabilised by six membered-ring intramolecular hydrogen bonding in poorly polar solvents, while *trans* or *cis* di-keto forms are usually stabilised by an increase in polarity and, to a smaller extent, by hydrogen-bond donating solvents [9,10]. The presence of both keto-enol and phenolic groups make curcumin a weak triprotic acid that is mostly present in its neutral form under physiological conditions (pH 7.4) as pK_a1_ is 8.54 [11]. Another interesting feature of the keto-enol group is its ability to act as a bidentate hard chelator with two coordination oxygens forming a six membered-ring when metal-coordination occurs. High affinity for the metal ion typically results in the drop in the keto-enol dissociation constant, and in past decades, curcumin has been demonstrated to be a suitable bidentate chelating agent for hard Lewis acids such as iron [11,12], gallium [13] and copper [14]. Particularly interesting are the most recent applications of curcumin in the development of metal-based hybrid materials for drug delivery [15,16] or metal detection [17].

Throughout recent decades, many curcumin derivatives have been designed, synthesised and investigated to unravel biological mechanisms, improve the efficacy/activity and enhance bioavailability. The latter represents one of the main goals for transforming curcumin into a therapeutic agent. Since 2017, a great number of investigations have focused on the anticancer and neuroprotective activities of curcumin and curcuminoids; in fact, among almost 17,000 review and research articles, 3700 are related to cancer and more than 1300 deal with neurodegenerative diseases. 

The studies that have investigated the anticancer activity included several tumours, as reported in up-to-date reviews [18,19,20,21,22]. One of the possible pathways through which curcumin inhibits cancer cell proliferation could be the regulation of certain noncoding RNAs. These sequences have been shown to exert critical roles in regulating cancer cell biology, becoming promising targets for anti-cancer treatments [20]. Curcumin was demonstrated to regulate multiple cell signalling pathways which are crucial in cancer development and progression, including NF-κB, STAT3, activated protein-1 (AP-1), epidermal growth response-1 (Egr-1) and p53 [23]. For instance, the use of curcumin nanoformulations in colorectal cancer was recently reviewed by Wong et al. [24]. Moreover, curcumin was demonstrated to induce apoptosis in vitro against triple negative breast cancer cells. This cancer subtype is considered the most invasive and aggressive among all breast cancers; in fact, it accounts for approximately 20% of all cases and represents about 50% of mortality [25,26].

Concerning neuroprotective activities, curcumin can both suppress the overexpression of inflammatory mediators via inhibiting the TLR4-MAPK/NF-B pathway and reduce neuronal apoptosis via a mechanism involving the TLR4/MyD88/NF-B signalling pathway in microglia/macrophages. Particularly, targeting TLR4 makes curcumin an important pharmacophore for the design of new therapies of neurological diseases [27]. Recent studies have shown that curcumin also possesses cognitive-enhancing properties that may help to delay or prevent neurodegenerative diseases, including Alzheimer’s disease [28]. In this exception, curcumin inhibits the formation and promotes the disaggregation of Aβ-amyloid plaques, attenuates the hyperphosphorylation of tau-protein and enhances its clearance, binds copper, lowers cholesterol, modifies microglial activity, inhibits acetylcholinesterase and mediates the insulin signalling pathway [29].

To overcome the curcumin instability and poor bioavailability, new formulations or drug delivery systems have been exploited. Recently, Small et al. reported a long-term trial highlighting how curcumin led to significant memory and attention benefits associated with decreases in plaque and tangle accumulation in brain regions modulating mood and memory when administered to nondemented adults in a bioavailable form (Theracurmin^®^) [30]. 

In this landscape, the possibility of obtaining diagnostic tools for the early-stage diagnosis of cancer or neurodegenerative diseases, harnessing the properties of curcumin-like structures, is particularly attractive. Hence, the development of curcumin-based radiotracers represents a useful approach not only to elucidate metabolic and pharmacokinetics aspects of promising molecules but also to obtain suitable pharmaceuticals for imaging purposes in nuclear medicine applications. A radiotracer is a drug that exhibits a selective affinity for an aberrant metabolic process or specific physiological targets such as those expressed, for instance, by tumour cells or neurodegenerative pathologies. In this molecular structure, a radioactive atom that exerts the function of a beacon for visualising the target process/product, is inserted. Nuclear medicine is a branch of medicine that utilises such radioactive compounds for diagnoses or therapies of diseases. It is important to underline that a radiotracer is used in such a low amount that no pharmacological activity is exerted by the molecular structure itself. In the specific case of radiolabelled curcumin derivatives, the only physiological effects recorded are those related to their radioactive emission. This review aims to provide an up-to-date overview of the recent achievements aiming to obtain radiopharmaceuticals based on curcuminoid structure.

## 2. Radiohalogenated Curcumin Derivatives

### 2.1. Labelling with Fluorine-18:

Thanks to its straightforward and quantitative production by means of low-energy medical cyclotrons, fluorine-18 (E_β,max_ = 634 keV, 109.8 min) is nowadays the most used positron-emitting radionuclide for nuclear medicine examinations [31]. Radiolabelling with fluorine-18 is usually performed on aliphatic precursors by nucleophilic substitution reactions [32]. The starting material for these kinds of reactions is the aqueous solution of [^18^F]F^−^ directly available from the cyclotron target after proper activation and purification. Actually, [^18^F]fluoride is recovered on anion exchange resins, activated with a phase transfer catalyst, such as tetraalkylammonium carbonates or aminopolyethers, and subsequently dried by azeotropic distillation with acetonitrile. Finally, the reaction proceeds in polar aprotic solvents according to an S_N_2-mechanism, with halides or sulphonic ester (namely mesylate, tosylate and triflate) acting as leaving groups.

**Figure 1:**
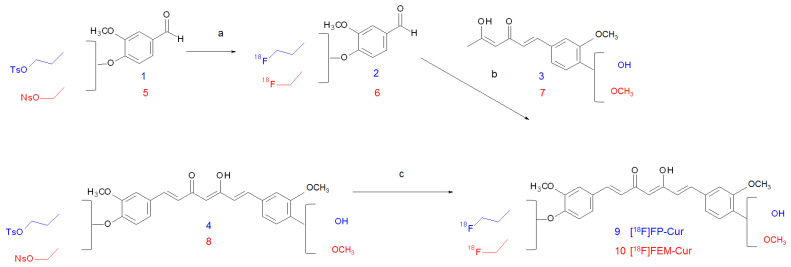
Reagents and conditions: (**a**) n-Bu_4_N[^18^F]F, CH_3_CN, 120 °C, 15 min; (**b**) B_2_O_3_, (n-BuO)_3_B, piperidine, ethyl acetate, 120 °C, 20 min, 0.4 N HCl, 90 °C, 5 min; (**c**) n-Bu_4_N[^18^F]F, THF, 95 °C, 20 min.

The first [^18^F]-labelled curcumin derivative was synthesised by Ryu and co-workers in 2006 with the purpose of developing a radiotracer able to visualise Aβ-plaque accumulation in the human brain [33]. The synthesis envisaged the addition of an alkyl chain containing the radionuclide to one of the curcumin phenolic groups. The labelling was first attempted by direct fluorination of the acetyl-protected tosylated precursor (**4**); however, due to the low yield (13%) and low specific activity obtained, a second route was pursued. The path entailed the reaction between compound (**1**) and activated [^18^F]F^−^, followed by aldol condensation of the product with the acetylacetone-boron complex of (**3**). After HPLC purification, the reaction afforded [^18^F]FP-Cur (**9**) with an overall decay-corrected RCY of 16–25% and a molar activity of 37.6 GBq/μol (Figure 1, blue route).

[^18^F]FP-Cur underwent affinity studies and showed a 2.9-fold higher binding affinity for Aβ aggregates than curcumin itself (Ki value of 0.07 vs. 0.20 nM). Moreover, a reasonably high initial brain uptake in normal mice (0.52% ID/g) followed by rapid washout (0.11% ID/g within 30 min) was found. However, biodistribution studies demonstrated high and persistent radioactivity accumulation in the liver and spleen as well. On the other hand, [^18^F]FP-Cur did not appear to undergo metabolic defluorination, but it was almost completely converted in a more polar metabolite within 60 min in the blood flow. This radioactive metabolite did not cross the BBB and partially gave reasons for the high radioactivity found in the liver and intestines.

Based on these results, the same group tried afterward to modulate the lipophilicity of [^18^F]FP-Cur by replacing a phenyl *para*-OH group with a methoxyl- group and/or by varying the propyl- chain of the linker with an alkoxyl- or a polyethoxyl- chain [34]. The aim of this study was to improve the brain permeability of fluorinated curcumin derivatives and consequently enhance their brain uptake. It was found that, in the new compounds, the binding affinity for Aβ-amyloid plaques was slightly decreased by pegylation of the linker (K*_i_* ranges from 3.01 to 4.65 nM). Conversely, methoxyl- substitution generally generated derivatives with higher affinities than the corresponding hydroxyl- substitution did. This last finding suggested that the presence of OH- groups on phenyl rings is not essential for avid binding to Aβ(1–42) aggregates.

Among all the tested derivatives, 1-(4-fluoroethyl)-7-(4′-methyl)curcumin (FEM-Cur) was the one with the highest affinity for Aβ-amyloid plaques, and it was shown to distinctively stain plaques in transgenic Tg APP/PS-1 mouse brain sections. The staining was performed on the “cold” compound by harnessing the natural fluorescence of curcumin derivatives. FEM-Cur was hence selected for the labelling with fluorine-18 and for further preclinical evaluation as a PET agent. The labelling reaction was carried out by following both methods previously reported for [^18^F]FP-Cur, and, in addition, the influence on the yield of a new precursor containing a nosylate group such as sulfonic acid ester was evaluated. In the first method, the direct labelling of **8** with n-Bu_4_N[^18^F]F^−^ afforded [^18^F]FEM-Cur (**10**) in an overall d.c. RCY of 10–17% in 60 min. The second method proved to be more time-consuming because it involved the nucleophilic addition of [^18^F]F^−^ to **5** followed by the aldol condensation of the product obtained with **7** and HPLC purification, but it yielded 15–25% and afforded the highest molar activity (37.6 GBq/µmol). The use of a nosylate moiety as a leaving group ameliorated a yield of 5–7% compared to the corresponding tosylated one in both approaches. Both synthetic pathways are shown in Figure 1, red route.

Pharmacokinetics and the tissue biodistribution of [^18^F]FEM-Cur in normal mice were then comparable to those of [^18^F]FP-Cur. At 2 min post-injection, a high amount of radioactivity accumulated in the lungs, liver and spleen but rapidly decreased over time. Conversely, the brain uptake of [^18^F]FEM-Cur was 2.7-fold higher than the uptake of [^18^F]FP-Cur at 2 min post-injection (1.44% ID/g) and grew to 4.1-fold at 30 min post-injection (0.45% ID/g). These results were probably related to the increased lipophilicity of [^18^F]FEM-Cur that allowed a higher BBB permeability. The authors concluded that the derivative had the potential to be a radiotracer for the imaging of Aβ-amyloid plaques, but further studies were warranted.

**Figure 2:**
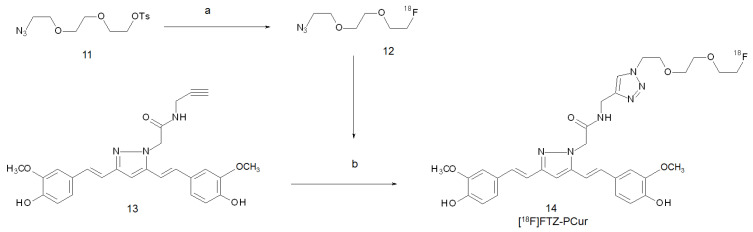
Reagents and conditions: (**a**) K_2_CO_3_, Kry2.2.2., [^18^F]F^−^, DMSO, 100 °C, 5 min; (**b**) CuSO_4_ (aq), sodium ascorbate (aq), DMSO, RT, 15 min.

It has been reported that curcumin has a poor bioavailability which may result from its rapid metabolism in the liver and intestinal walls [35]. Moreover, it exhibits a limited solubility in physiological media and low stability in vivo due to the high reactivity of its keto-enol moiety. Likewise, radiotracers based on the curcumin structure share these limitations, and their biodistribution is flawed by the rapid metabolism of the backbones. For these reasons, Rokka and co-workers converted the keto-enol moiety to a pyrazole ring through the reaction with a hydrazine-derivative compound, and they tried to study the performances of this derivative when labelled with fluorine-18 [36]. Pyrazole curcumins were previously found to be extremely stable at physiological pH and were suggested to be good potential replacements for curcumin in future drug development [37]. The labelling was pursued by exploiting a copper (I)-catalysed azide-alkyne cycloaddition between the pyrazole curcumin precursor (**11**) and 2-[2-(2-azidoethoxy)ethoxy]ethyl-[^18^F]fluoride (**12**) (Figure 2). The alkyne-ending chain was tethered to one of the nitrogen atoms of the pyrazole ring. The reaction was performed one-pot and product **14** (namely [^18^F]FTZ-PCur) was obtained in a 21 ± 11% yield, molar activity > 1 TBq/mmol, and RCP > 99.3% after semi-preparative HPLC purification.

The [^18^F]FTZ-PCur capability of binding Aβ-amyloid plaques was evaluated in vitro on brain cryosections of transgenic APP23 and wild-type mice. APP23 mice are known to develop an extensive Aβ-amyloid pathology, and the first Aβ deposits are usually observed at six months of age [38]. [^18^F]FTZ-PCur accumulation was compared with thioflavin-S staining, and displacement tests were conducted with the addition of several concentrations of PIB. The in vivo biodistribution of [^18^F]FTZ-PCur was studied on the same two animal models through a 60 min dynamic PET scan. The radiotracer showed specific accumulation on Aβ-amyloid plaques in vitro, but very low brain accumulation in vivo was detected and no differences were highlighted between the pharmacokinetics of the two animal models. Moreover, [^18^F]FTZ-PCur exhibited a relatively low stability in mice blood, accounting for only 45% of intact radiotracer after 10 min of injection. The authors concluded that, in spite of promising results in vitro and the fast blood clearance, low BBB penetration and low stability are strong drawbacks for the successful detection of Aβ-amyloid deposition in vivo. Thus, more studies are needed on this class of derivatives to overcome these issues.

**Figure 3:**
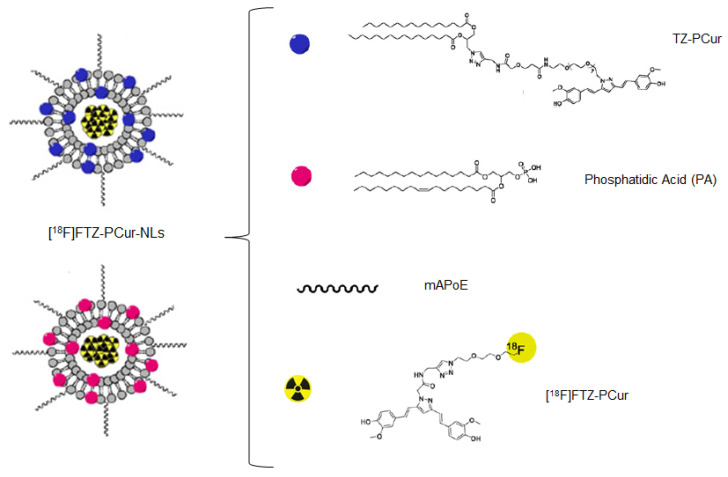
Scheme of functionalised NLs embedding [^18^F]FTZ-PCur derivative. The figure has been obtained by modifying Figure 1 in reference [39]. Reprinted with permission from [39]. Copyright 2016 Elsevier.

As a result, the following couple of years, the same authors attempted an innovative approach based on the encapsulation of the [^18^F]FTZ-PCur derivative in functionalised nanoliposomes (NLs) [39]. NLs can enhance drugs delivery to target tissues and protect the encapsulated compound from metabolic decomposition [40,41]. Functionalisation of the NLs surface was performed by tethering anionic phospholipids (PA) or lipid-TZ-PCur derivatives, as well as a monomeric derivative of ApoE peptide residue 141–150 (mApoE). In fact, while BBB penetration is usually challenging for naked nanoparticles, mAPoE addition was shown to increase the BBB penetration in a cell culture model and in experimental animal studies [42,43]. In addition, functionalisation with anionic phospholipids or lipid-curcumins have been shown to yield NLs able to bind Aβ(1–42) fibrils in vitro [44,45]. The preparation of functionalised NLs was carried out by thin-film hydration, extrusion, and gel chromatography technologies. The following encapsulation of [^18^F]FTZ-PCur was achieved by using a solution of the radiotracer as a rehydration solution. A schematic representation of the NLs structure herein described is depicted in Figure 3.

**Figure 4:**
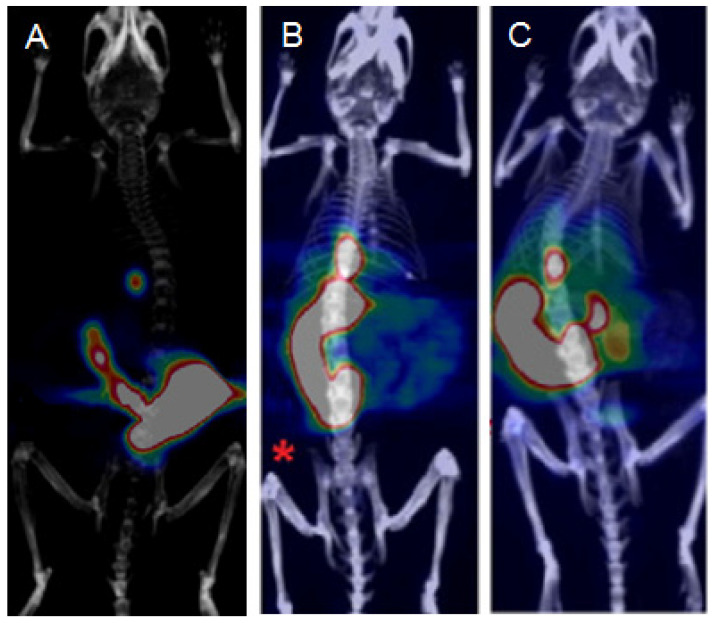
In vivo PET/CT images presenting the whole body biodistribution of [^18^F]FTZ-PCur (**A**), PA-mApoE-[^18^F]FTZ-PCur-NLs (**B**), and TZ-PCur-mApoE-[^18^F]FTZ-PCur-NLs (**C**). Figures present summed PET images taken 30–60 min after injection into a wild-type (WT) mouse. Figure has been obtained by merging and elaborating images drawn from references [36,39]. Reprinted with permission from [36,39]. Copyright 2016 Elsevier.

Unfortunately, the studies in WT and transgenic Aβ-plaques-developing mice have shown how the biodistribution of both liposomal formulations reflected the biodistribution of [^18^F]FTZ-PCur alone (Figure 4). In both cases, the initial uptake was seen in the liver and it was followed by high accumulation in the intestine. Radioactivity in the brain was very modest and no significant difference was found between the two animal models. Further preclinical evaluations showed that the liposomal formulations exhibited a certain instability that brought a consequent [^18^F]FTZ-PCur release to the NLs leakage. This was likely the reason for the similar in vivo behaviour between the NLs formulations and the free radiotracer itself.

**Figure 5:**
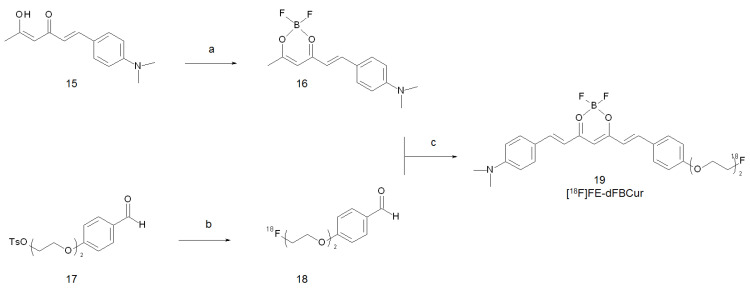
Reagents and conditions: (**a**) BF_3_·Et_2_N, CH_2_Cl_2_, RT, 3 h; (**b**) n-Bu_4_N[^18^F]F, CH_3_CN, 110 °C, 10 min; (**c**) n-butylamine, ethyl acetate, 110 °C, 20 min.

Due to the poor results obtained with pyrazole-curcumin derivatives in terms of BBB penetration so far, an alternative modification of the original FP-Cur and FEM-Cur compounds was explored by Kim et al. in 2019 [46]. The derivatisation envisaged the complexation of a di-fluoroboron group to the 3,5-diketone moiety and the replacement of the phenyl ring substituents with several methylamino groups. The modifications had the additional result of shifting the excitation and emission wavelengths of the new compounds to 540 and 640 nm, respectively [47]. Hence, unlike with curcumin, these derivatives could be used as an optical imaging probes as well. The compound showing the best performance in terms of Aβ-aggregates affinity in vitro (K_d_ = 19.66 nM) was selected for subsequent labelling with fluorine-18. Synthesis was carried out following a pathway similar to that reported previously for [^18^F]FP-Cur and [^18^F]FEM-Cur, as resumed in Figure 5.

[^18^F]FE-dBFCur (**19**) was obtained after HPLC purification in a 17–22% d.c. overall yield with a molar activity of 39–46.8 GBq/µmol. Biodistribution studies at 60 min post injection (p.i.) showed high radioactivity accumulation in the liver, spleen, and small intestine of normal mice. Brain uptake was poor, although the computed partition coefficient (2.58) should have been suitable for crossing the BBB. A starting value of 0.49% ID/g at 2 min p.i. was reported to rise up to achieve a maximum of 1.19% ID/g at 60 min p.i. On the other hand, metabolic studies on brain homogenates have attested that [^18^F]FE-dBFCur disappeared almost entirely within 60 min p.i, and new polar radioactive peaks appeared at the origin of radio-TLC scans. Hence, the growth of activity in mice brain overtime was likely due to the generation and retention of polar radioactive metabolites. Authors argued that the in vivo characteristics of [^18^F]FE-dBFCur were distinct from those of the ^18^F-labelled curcumin derivatives studied so far. In fact, [^18^F]FP-Cur and [^18^F]FEM-Cur had poor brain permeability at 2 min p.i. and showed rapid wash-out within 30 min. Moreover, these radiotracers remained intact once they were taken up [33,34].

**Figure 6:**
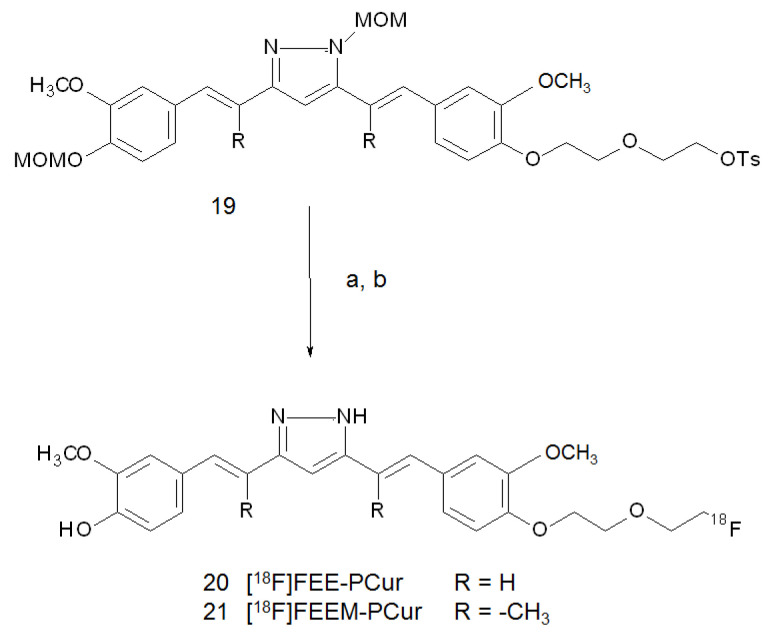
Reagents and conditions: (**a**) n-Bu_4_N[^18^F]F, CH_3_CN, 110 °C, 10 min; (**b**) 6 N HCl, 120 °C, 10 min.

Another possible application of radiolabelled curcumin and curcumin derivatives might concern the visualisation of tumours. Actually, a noticeable anticancer activity in vitro was attested by using this family of compounds over the years [18,19,20,21,22,48]. In particular, it was reported that pyrazole-curcumin is more effective than curcumin itself for the prevention of hepatocarcinogenesis in rats [49]. On this basis, Shin and co-workers synthesised a series of pyrazole-curcumin derivatives, elucidated their metabolism and evaluated the two of them with the highest anti-tumoural activity as imaging agents in HUVEC and C6 glioma cells [50]. The [^18^F]-labelling was performed by nucleophilic substitution of the MOM-protected pyrazole-curcumin tosylated precursors (**19**), followed by cleavage of the products under strong acid conditions. The conversion of the keto-enol moiety to the pyrazole ring was previously obtained by reaction with hydrazine hydrate. Final HPLC purification gave [^18^F]FEE-PCur (**20**) and [^18^F]FEEM-PCur (**21**) in overall d.c. radiochemical yields of 25–35% and with specific activities of 51.06 and 44.5 GBq/μmol, respectively. Structures of these derivatives are shown in Figure 6.

**Figure 7:**
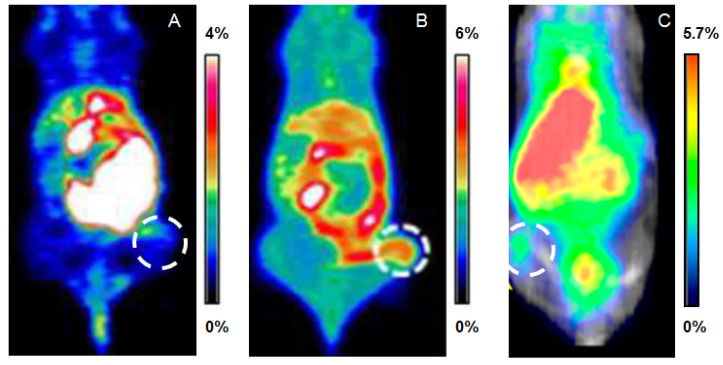
In vivo PET images at 65 min of [^18^F]FEE-PCur (**A**) and [^18^F]FEEM-PCur (**B**) in C6 glioma-bearing mice, and at 60 min of [^68^Ga]Ga-DOTA-C21 in a HT29 colorectal tumour-bearing mouse (**C**). Figure has been obtained by merging and elaborating images from references [50,51]. Reprinted with permission from [50,51]. Copyright 2011 RSC Publishing.

The radiotracers were then injected in mice bearing C6 glioma xenografts, and microPET images followed by biodistribution studies were acquired. Both the derivatives demonstrated high accumulation in the intestines, but [^18^F]FEEM-PCur exhibited a significantly higher tumour uptake than [^18^F]FEE-PCur at 65 min p.i. (3.2% and 0.98% ID/g, respectively). On the other hand, tumour-to-background ratios were within the same ranges, and the small intestine uptake was markedly higher for [^18^F]FEE-PCur, indicating a more rapid clearance. A paradigmatic comparison of the two radiotracers is shown in Figure 7A,B. The authors concluded that microPET and biodistribution studies demonstrated the potential of these probes for the imaging of C6 tumours in mice, but further studies are needed to shed light on their molecular targets.

### 2.2. Labelling with Iodine-125

Iodine exhibits at least four relevant radionuclides that have been extensively used in biological science and in nuclear medicine. Iodine-131 is a β^−^-emitting isotope with a maximum energy of 606 keV (89%) and a half-life of 8.0 days. Beta radiation has optimal therapeutic efficacy as it penetrates the tissues from 0.6 to 2.0 mm from the site of uptake. It is a γ-emitter as well, but the high energy of the photons emitted (364 keV, 81%) renders this radionuclide quite unsuitable for imaging purposes [52]. Iodine-124 is a β^+^-emitting isotope (25.6%) with a half-life of 4.18 days. It can be produced through numerous nuclear reactions by means of a medical cyclotron, warranting a stable source of radionuclides in nuclear medicine units [53]. The gamma-emitting isotopes iodine-123 (half-life 13.1 h, 159 keV, 87%) and the longer-lived and less energetic iodine-125 (half-life 59.4 days, 35 keV) are both used as nuclear imaging tracers to evaluate anatomic and physiologic functions. Due to the low-energy radiation and the long half-life, iodine-125 is the most suitable isotope to serve as a surrogate of all the other iodine radionuclides in pre-clinical research and drugs development. Labelling with iodine-125 normally involves incorporation of the radionuclide into tyrosyl- or histidyl-like residues belonging to the molecular structure of interest [54].

**Figure 8:**
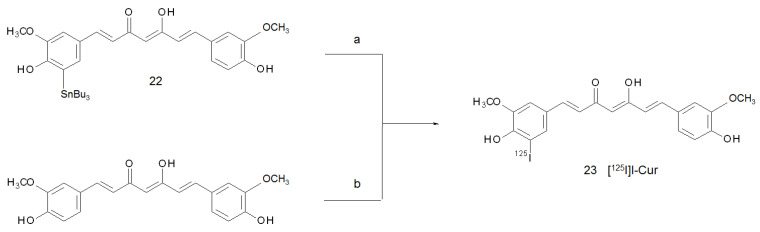
Reagents and conditions: (**a**) Na[^125^I]I, 1 N HCl, 3% H_2_O_2_, 10 min; (**b**) Iodogen, CHCl_3_, Na[^125^I]I, RT, 10 min (the product obtained with this path is an assumption of the authors of this review).

The first [^125^I]-iodinated curcumin derivative was prepared in 2006 by Ryu and co-workers with the aim of obtaining a suitable tool for monitoring the formation of Aβ plaques in the brain [33]. The radiotracer was synthesised by radioiododestannylation of the corresponding tributylstannyl precursors with Na[^125^I]I (**22**). The reaction was quenched with saturated NaHSO_3_, and the mixture was purified by HPLC. [^125^I]I-Cur (**23**) was obtained with a d.c. radiochemical yield and molar activity of 35–40% and 87.3 GBq/µmol, respectively (Figure 8, route a). Unfortunately, the introduction of an iodine atom into a methoxy-phenolic ring was found to decrease significantly the in vitro binding affinity of these kinds of derivatives for Aβ-aggregates, and the look for a radio-iodinated curcumin devoted to imaging Alzheimer’s disease was not pursued further. However, in 2016, Kumar and co-workers reported the synthesis of [^125^I]-iodinated derivatives as a tumour-targeting probe [55]. The radiotracer was obtained through a direct iodination of curcumin using the Iodogen method [56]. Although the structure was not reported in the original paper, the molecule obtained was likely the mono-substituted [^125^I]I-Cur derivative. The pathway envisaged the oxidation of [^125^I]I^-^ by 1,3,4,6-tetrachloro-3α,6α- diphenylglycoluril and the consequent addition to the curcumin tyrosyl-like group (Figure 8, route b). After extraction with chloroform, a RCY of 75% and a RCP > 95% were reported, respectively.

The biodistribution of [^125^I]I-Cur was then assessed in lymphoma-bearing C57BL/6 mice. The examination attested high accumulation in all major organs (stomach, intestine, liver, kidneys, lung and spleen) and a constant tumour uptake of 3.3% ID/g in the first 60–180 min that was gradually washed out in 48 h. The clearance in blood was also slow and a gradual increase in the thyroid activity suggested partial in vivo de-iodination of the radiotracer. Authors concluded that [^125^I]I-Cur has potential for the specific targeting of lymphomas, although further modification of the structure is needed to improve the in vivo pharmacokinetics.

## 3. Curcumin Derivatives Labelled with Metal Radionuclides

### 3.1. Labelling with Technetium-99m

Technetium-99m is a low-energy γ-emitting radioisotope (Eγ = 140 keV, 89%) with a moderate half-life of 6 h. The emission is well suited to be detected by medical equipment such as SPECT cameras. The most attractive feature of this radionuclide is that it is produced by a commercial transportable generator through the decay of its long-lived parent radionuclide molybdenum-99 (66 days half-life). Nuclear medicine services can nowadays easily supply the generators needed for covering their clinical preparations on weekly bases. Thus, technectium-99m is the most commonly used radionuclide in nuclear medicine examination so far [57]. Recently, the interruption of the global supply chain of reactor-produced molybdenum-99 has also forced the scientific community to investigate alternative production routes of technetium-99m, and the production with cyclotrons has also been considered [58,59]. However, the use of generators remains the most common way for providing this radionuclide.

Generally, the preparation of a [^99m^Tc]-labelled radiotracer involves the chemical reduction of the [^99m^Tc]TcO_4_^−^ obtained from the generator and the consequent complexation with proper ligands. The nature and amount of the reductive agent, as well as of the ligands, can generate sundry oxidation forms (from −1 to +7) and chemical *cores*. The radiochemistry of technetium-99m has been extensively studied over the years, bringing forth a plethora of possible chemical pathways for the labelling and a consequent large number of radiotracers as well [60].

**Figure 9:**
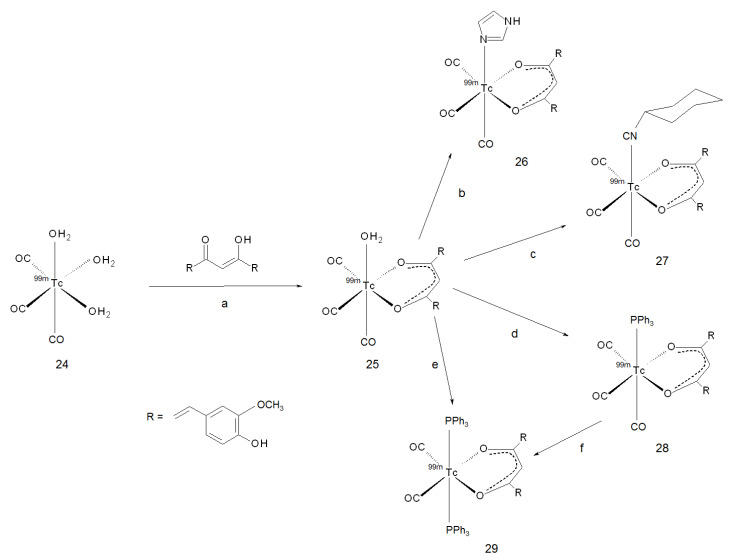
Reagents and conditions: (**a**) curcumin, DMSO 70 °C, 15 min; (**b**) imidazole, DMSO, 70 °C, 30 min; (**c**) isocyanocyclohexane, DMSO, RT, 30 min (**d**) triphenylphosphine, MeOH, RT, 20 min; (**e**) triphenylphosphine, MeOH, 60 °C, 20 min; (**f**) triphenylphosphine, MeOH, 60 °C, 20 min.

In 2011, Sagnou and co-workers were the first group to study [^99m^Tc]-curcumin mixed ligand complexes with a tricarbonyl-*core*, in the form *fac*-[^99m^Tc(CO)_3_(curcumin)(L)] (where L = imidazole or isocyanocyclohexane). The formation of the [^99m^Tc(CO)_3_(OH_2_)_3_]^+^ *core* (**24**) was first obtained by the reaction of [^99m^Tc]TcO_4_^−^ with a kit containing NaBH_4_, Na_2_CO_3_ and Na-K tartrate, as described in the literature [61]. Then, curcumin and L, each dissolved in the proper solvent, were added sequentially, and the mixture was mildly heated up to obtain the final “2 + 1” neutral complex (**26** and **27,** respectively). A couple of years after, the same group achieved the formation of two additional complexes by following an analogue pathway [62]. The first complex was structurally analogue to the previous ones but with L = triphenylphosphine (**28**), and the second was in the form *cis*-*trans*-[^99m^Tc(CO)_2_(curcumin)(L)_2_] (**29**). Generally, the formation of the *core* and the first intermediate containing curcumin as a *OO* bidentate ligand (**25**) proceeded in high yield (>90%). Conversely, the replacement of the water molecule by L was more difficult, and its success depended on the ligand. The reported yields were 25–35% for imidazole (**26**) and >90% for isocyanocyclohexane (**27**). In the case of triphenylphosphine, complex **28** was obtained in 80% yield when the mixture was reacted at room temperature, and complex **29** was obtained quantitatively when it was heated at 60 °C. A global panel of the reactions herein described is resumed in Figure 9.

Following similar pathways, the corresponding rhenium complexes were synthesised as well, allowing deeper structure characterisation through classical chemical techniques such as mass spectrometry, NMR and IR spectroscopy, and X-ray analysis. Eventually, the capability of binding Aβ-amyloid plaques of the rhenium complexes was tested by staining human *post-mortem* AD-diagnosed brain sections. The study was performed by harnessing the natural fluorescence of curcumin and its derivatives. All the complexes proved to bind selectively to the plaques, allowing clear visualisation of both diffused and dense ones, as attested by comparison with thioflavin-S staining. These results hinted that curcumin complexes can retain and potentially enhance the original affinity of curcumin for Aβ-amyloid plaques. The authors speculated that these findings paved the way to further studies in which curcumin acts as a bidentate ligand in a mixed pharmacophore technetium-99m complex; however, unfortunately, no additional studies on the behaviour of these complexes in vivo have been reported so far.

### 3.2. Labelling with Gallium-68

**Figure 10:**
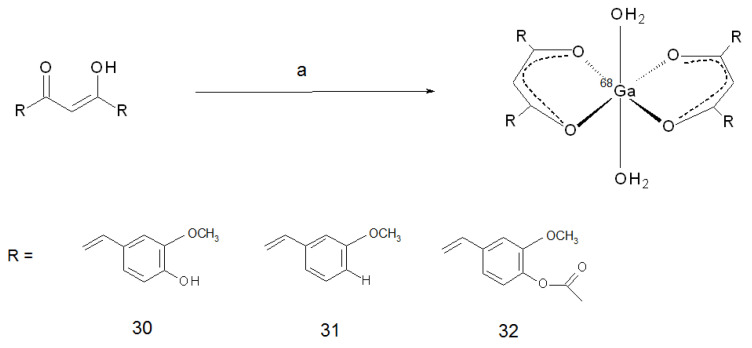
Reagents and conditions: (**a**) ^68^Ga^3+^ (0.05 M HCl), 1.5 M CH_3_COONa (pH 5), 95 °C, 5 min.

Gallium-68 (E_β,max_ = 1899 keV, 89%, 67.7 min) is the most valuable new radionuclide that has achieved a steady utilisation in clinical practice in recent years. The reason for its widespread use is mainly due the fact that it can be obtained from a commercial transportable generator by the decay of its parent radionuclide germanium-68 (271 days half-life) [63]. Due to the long half-life of germanium-68, the ^68^Ge/^68^Ga generator guaranteed a reliable gallium-68 source for a long life span, making the manufacturing of positron-emitting radionuclides independent of the presence of a medical cyclotron at the site of production. Thank to this feature, the last decade has been characterised by a flowering of research on gallium-68-radiolabelled compounds that conveyed to clinical utilisation some interesting radiotracers, such as radiolabelled somatostatin analogues and PSMA inhibitors [64,65]. Coping with the increasing requests, recently, the production of gallium-68 with accelerators by irradiation of a solid target of enriched zinc-68 or of a concentrated solution of zinc-68 salt was reported as well [66,67,68]. From a chemical point of view, Ga(III) is a hard Lewis acid forming preferentially hexacoordinated complexes with O and N donor atoms. The first [^68^Ga]-labelled curcumin derivatives were reported by Asti and co-workers [69] and were obtained in the form of homodimeric monocationic complexes. The complexes formation leveraged the coordinating properties of the keto-enol moiety of curcumin and curcuminoids. Radiotracers (namely, [^68^Ga](CUR)_2_^+^ **30**, [^68^Ga](bDHC)_2_^+^ **31**, and [^68^Ga](DAC)_2_^+^ **32**) were obtained almost quantitatively by adding 60 nmol of precursor to a portion of gallium-68 generator eluate, in the presence of a pH 5 buffering agent, and heating the mixture for 5 min at 95 °C (Figure 10). The characterisation was carried out on the corresponding synthesised stable compounds, and a 2:1 ligand-to-metal stoichiometry was attested. The curcuminoids occupied the four equatorial positions of a theoretical octahedron, while two solvent molecules completed the sixfold coordination in apical positions.

The following years, the same group was devoted to rehearse the binding affinity for Aβ-amyloid plaques and the uptake in sundry cancer cell lines of these [^68^Ga]-curcuminoid complexes [70,71]. Concerning the Aβ-amyloid plaques, the investigation was performed on synthetic fibrils and on brain sections of Tg2576 mice by harnessing the natural fluorescence of the curcumin derivatives. Tg2576 mice are animal-developing Aβ-amyloid tangles starting from 5–6 months of age and progressing until 12–14 months of age [72]. These studies attested that all compounds were able to visualise synthetic fibrils and Aβ-amyloid plaques on the mice brain section when the samples were stained with a solution thereof. However, Aβ-amyloid plaques could not be visualised on brain sections after in vivo injection of the compounds. The result was likely due to the low stability of the complexes in vivo and to the challenging passage through the BBB that hampered their accumulation in mice brain [70].

Upon the possibilities of using these derivatives as potential radiotracers for imaging cancer, the uptake of [^68^Ga](CUR)_2_^+^, [^68^Ga](bDHC)_2_^+^ and [^68^Ga](DAC)_2_^+^ was evaluated in K562 (chronic myelogenous leukaemia), HT29 (colorectal adenocarcinoma), A549 (lung carcinoma), PC3 (prostate cancer) and MDA-MB-231 (breast adenocarcinoma) cell lines, and it was compared to the uptake of peripheral blood mononuclear cells (PBMC) [71]. It was found that [^68^Ga](CUR)_2_^+^ and, to a higher extent, [^68^Ga](DAC)_2_^+^ showed a preferred uptake in the HT29 colorectal cancer cell line and K562 lymphoma cell line with respect to normal human lymphocytes, hinting a possible application in the imaging of these tumours.

**Figure 11:**
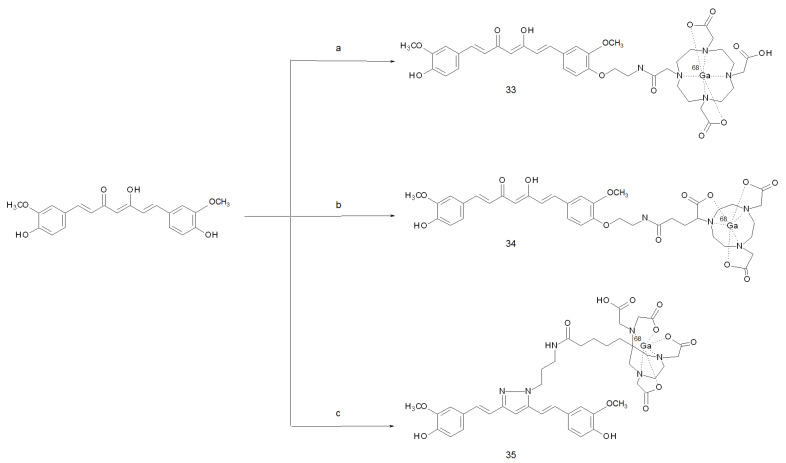
Reagents and conditions: (**a**) 1, 2-(Boc-amino)ethyl bromide, K_2_CO_3_, DMF, RT, 48 h, 2, TFA/DCM 2/8, RT, 30 m, 3, DOTA-NHS ester, DIPEA, DMF, 1 h, 4, ^68^Ga^3+^ (0.05 M HCl), 1.5 M CH_3_COONa (pH 5), 95 °C, 5 min; (**b**) 1, 2-(Boc-amino)ethyl bromide, K_2_CO_3_, DMF, RT, 48 h, 2, TFA/DCM 25/75, RT, 30 m, 3, NODAGA(tBu)_3_, HBTU, DIPEA, DMF, 1 h, 4, 80% TFA/DCM, RT, 3 h, 5, ^68^Ga^3+^ (0.05 M HCl), 1.5 M CH_3_COONa (pH 5), 95 °C, 5 min; (**c**) 1. tBuHPC, CH_3_COOH, reflux, 6 h, 2, TFA/DCM 25/75, RT, 30 m, 3, AAZTA-HBTU ester, DIPEA, DMF, RT, 8 h, 4, TFA/TIS/H_2_O 90/5/5, RT, 2 h, 5, ^68^Ga^3+^ (0.05 M HCl), 1.5 M CH_3_COONa (pH 5), RT, 5 min.

Based on these findings, a new series of compounds in which gallium-68 was stably coordinated by several bifunctional chelators (namely, DOTA, NODAGA and AAZTA) tethered to a curcumin-based molecular vector was developed [51,73]. Synthesis of these derivatives generally entailed a first step in which the curcuminoid was functionalised with a protected spacer able to bind the chelator upon cleavage of the protecting group. After the coupling reaction, the pathway proceeded with the de-protection of the chelator itself and the complexation with gallium-68. The needing of gel column chromatography and HPLC purifications among the steps made the synthesis of the precursor cumbersome and low-yielding (5 to 10%). On the other hand, the labelling was almost quantitative and no further purification was necessary. Through this method, the radiotracers [^68^Ga]Ga-DOTA-C21 (**33**), [^68^Ga]Ga-NODAGA-C21 (**34**) and [^68^Ga]Ga-AAZTA-PC21 (**35**) were obtained. An overview of these [^68^Ga]-curcuminoid derivatives and the steps involved in their synthesis is given in Figure 11. All the compounds exhibited much higher stabilities *in serum* if compared to the previous ones in the order [^68^Ga]Ga-AAZTA-PC21 > [^68^Ga]Ga-NODAGA-C21 ≈ [^68^Ga]Ga-DOTA-C21.

[^68^Ga]Ga-DOTA-C21 underwent further investigations and its accumulation in HT29 colorectal cancer cells was found to be specific and time-dependent. Imaging and biodistribution studies on HT29 tumour-xenograft mice were also performed, and a tumour accumulation of 3.08 ± 0.53% ID/cc at 1 h post-administration was reported. [^68^Ga]Ga-DOTA-C21 appeared to have both renal and hepatic clearance with an uptake of around 6% and 12% ID/cc, respectively. Radioactivity in the heart, intestine, and lungs was high as well. Comparison among the biodistribution of [^68^Ga]Ga-DOTA-C21 and the [^18^F]-fluorinated derivatives [^18^F]FEE-PCur and [^18^F]FEEM-PCur can be estimated in the PET images of Figure 7.

### 3.3. Labelling with Scandium-44

Scandium-44 is a β^+^-(E_β,max_ = 1474 keV, 94%)- and γ-(E_γ_ = 1157 keV, 99%)-emitting radionuclide that decays to stable calcium-44 with a half-life of 3.97 h. It can be obtained as a daughter radionuclide of long-lived titanium-44 (t_1/2_ 60.4 a) from a ^44^Ti /^44^Sc generator or produced by the nuclear reaction of ^44^Ca(p, n)^44^Sc in small cyclotrons [74,75]. Due to the long half-life of titanium-44, the generator provides a cyclotron-independent source of scandium-44 for several decades, but it is not commonly available and can be supplied for research purposes only. From a chemical point of view, scandium behaves like a typical transition metal with a predominant oxidation state of +3. Likewise gallium, scandium is a hard Lewis acid and forms stable complexes with the same chelators. Scandium-44 is attractive for nuclear medicine applications as it has an almost four-times longer half-life and higher β^+^ branching than the most commonly used gallium-68. For this reason, it can be used for more accurate planning and dosimetry calculations, being able to cover imaging periods longer than a single day [76]. Along with gallium-68, the labelling of the curcumin derivatives NODAGA-C21 and AAZTA-PC21 with scandium-44 was also attempted by Orteca et al. [73]. The reactions were performed as described for gallium-68, but it was reported that the complexation kinetics of NODAGA-C21 with scandium-44 were quite slow and poorly reproducible (incorporation <50% after 30 min of incubation at 95 °C). On the other hand, [^44^Sc]Sc-AAZTA-PC21 was obtained quantitatively after 10 min of reaction at room temperature. The stability of this latter compound was remarkable (>90% after 8 h of incubation in PBS or HS and around 75% and 60% after 2 and 8 h in HB, respectively), but no further evaluation of the performances in vivo was carried out for this radiotracer.

## 4. Deuterium and Tritium-Labelled Curcumin Derivatives

**Figure 12:**
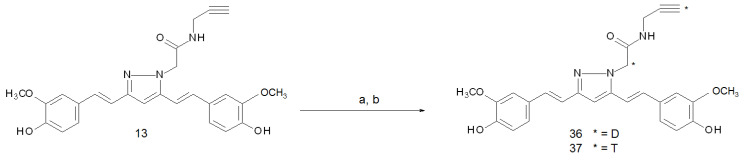
Reagents and conditions: (**a**) n-BuLi, iPr_2_NH, THF dry, 0 °C to RT (**b**) [^2^H]H_2_O or [^3^H]H_2_O, 0.5 h.

Deuterium (hydrogen-2) and tritium (hydrogen-3) are heavier isotopes of hydrogen that contain one and two neutrons, respectively. Deuterium is a stable isotope with a very low natural abundance (<0.02%) that can be obtained as a component of heavy water molecules by filtration or distillation of ordinary water in industrial implants [77]. Tritium is a radioactive nuclide with a 12.3 year half-life that decays to helium-3 emitting β^−^-particles with an average energy of 5.7 keV. It can be produced in nuclear reactors by the neutron irradiation of lithium-6 or boron-10 [78,79]. From a chemical point of view, bonds involving deuterium and tritium are, to some extent, stronger than the corresponding ones involving hydrogen. This difference is enough to elicit significantly slow kinetics in the biological processes involving deuterated and tritiated compounds. For this reason, both the isotopes are used as labels for tracing metabolic processes or chemical pathways. Deuterium can be distinguished from ordinary hydrogen most easily by its mass, using mass spectrometry or infrared spectrometry. Detection of tritium-labelled compounds is rather achieved by using liquid scintillation counting. On the other hand, the low energy of its emission makes this radionuclide useless for any kind of imaging or therapeutic purpose. In 2011, Zona and co-workers reported the labelling with deuterium and tritium of a pyrazole-curcumin derivative with the purpose of studying its trafficking trough the BBB in an in vitro model [80]. This research was a preliminary step for the development of the fluorine-18-labelled radiotracers aiming to detect Aβ-peptide accumulation in the brain [36,39], as already mentioned in Section 2.1. The deuterated compound (**36**) was obtained by reacting precursor **13** with lithium di-iso-propyl amide and subsequently with a 20-fold molar excess of [^2^H]H_2_O. The treatment afforded the isotopic exchange of both the protons in α to the amide position and of the acetylene proton with an overall yield of 88%. The replacement with tritium was performed in the same way (by using [^3^H]H_2_O, 2.5 mCi/mL), but the product (**37**) was further purified for eliminating radionuclides linked to the phenolic hydroxyl group of the two ferulic moieties (yield: 85%, molar activity: 405 mCi/mmol). An overview of the labelling reaction conditions is given in Figure 12.

## 5. Considerations and Conclusions

Several radiolabelled curcumin and curcuminoid derivatives have been studied over the years with the main goals of providing sensible radiotracers for the early diagnosis of Alzheimer’s disease and for the detection of neoplastic lesions by means of nuclear medicine instrumentations. Concerning the first application, although almost all the radiolabelled derivatives exhibited good affinity for Aβ-amyloid plaques in vitro, none of them proved to be a suitable tool for the imaging of Alzheimer’s disease in vivo. The main reason for this failure is likely due to the fact that the modification to the curcumin backbone needed for the insertion of a radionuclide decreases the lipophilicity of the structure, thwarting the trafficking through the BBB. This concept is particularly true when the labelling is performed with metal radionuclides as the chelators needed for exerting strong bonds with the radiometal introduce many hetero-atoms to the structure. The use of organic molecules-ubiquitous carbon-11 radionuclide (β^+^ emitter, E_β,max_ = 960 keV, 99.8%, 20.4 min) could result in lesser modifications or no modifications at all to the curcumin structure, but the labelling with this radioisotope has not been attempted so far. On the other hand, curcumin has a very low stability in physiological media, and modifications are recommended to elicit a pharmacological function. These two controversial needs are difficult to solve; hence, further findings are warranted for fostering this field of research. Concerning the application as radiotracers for the diagnosis of tumours, the curcumin derivatives that have been tested in vivo so far were clearly able to visualise the lesion but unfortunately showed a widespread distribution in many organs such as the liver, kidneys, spleen and lungs. The main issue for improving the performances of these radiotracers is due to the fact that the mechanism of their preferred uptake in some cancers has not been elucidated so far. This finding, along with the high accumulation in many organs, hampers the direct use of these radiolabelled derivatives as diagnostic tools, but the studies performed hitherto lay the foundation for developing further curcumin-based compounds with improved characteristics.

## Data Availability

Not applicable.

## Abbreviations

n.c.a.	No carrier added
PET	positron emission tomography
THF	tetrahydrofuran
HPLC	high-performance liquid chromatography
RCY	radiochemical yield
BBB	blood–brain barrier
d.c.	Decay-corrected
RCP	radiochemical purity
DMSO	dimethyl sulfoxide
WT	wild type
CT	computed tomography
TLC	Thin-layer chromatography
SPECT	single-photon-emission computed tomography
AD	Alzheimer’s disease
PSMA	Prostate-specific membrane antigen
DOTA	1,4,7,10-tetraazacyclododecane-1,4,7,10-tetraacetic acid
NODAGA	1,4,7-triazacyclononane,1-glutaric acid-4,7-acetic acid
AAZTA	1,4-bis(carboxymethyl)-6-[bis(carboxymethyl)]amino-6-methylperhydro-1,4-diazepine
DMF	dimethylformamide
DIPEA	*N,N*-diisopropylethylamine
TFA	trifluoroacetic acid
DCM	dichloromethane
HBTU	(2-(1*H*-benzotriazol-1-yl)-1,1,3,3-tetramethyluronium hexafluorophosphate
TIS	triisopropyilsilane
tBuHPC	tert-butyl bromopropylamine carbamate
PBS	phosphate-buffered saline
HS	human serum
HB	human blood
